# Ammonia Exposure Elevated 5-HT Expression, Reprogrammed Transcriptome and Microbiota Community in Yellow Catfish (*Pelteobagrus fulvidraco*) Gill During Early Ontogeny

**DOI:** 10.3390/microorganisms14040912

**Published:** 2026-04-17

**Authors:** Yuqing Jian, Kexin Xiong, Jiahong Zou, Xinyue Du, Shihao Liu, Yaoqiang Yue, Jian Gao, Wenjie Guo, Qingchao Wang

**Affiliations:** Key Laboratory of Aquacultural Facility Engineering (Ministry of Agriculture and Rural Affairs), College of Fisheries, Huazhong Agricultural University, Wuhan 430070, China; jianyuqing0618@webmail.hzau.edu.cn (Y.J.); xiongkexin@webmail.hzau.edu.cn (K.X.); zjiahong@webmail.hzau.edu.cn (J.Z.); duxinyue@webmail.hzau.edu.cn (X.D.); lsh000127@webmail.hzau.edu.cn (S.L.); yueyaoqaing@webmail.hzau.edu.cn (Y.Y.); gaojian@mail.hzau.edu.cn (J.G.); guowenjie@mail.hzau.edu.cn (W.G.)

**Keywords:** ammonia, *Pelteobagrus fulvidraco*, 5-HT, gill microbiota

## Abstract

The accumulated ammonia within the recirculating aquaculture systems threaten fish health, while little is known about the influences during early fish ontogeny. Using larval and juvenile yellow catfish (*Pelteobagrus fulvidraco*) as a model, a comprehensive experiment exposing fish to varying total ammonia nitrogen concentrations (0, 10, 20 mg/L for larvae; 0, 25, 125 mg/L for juveniles) was conducted to evaluate the effects on gill transcriptome and microbiota along with the serotonergic regulation. First, the serotonin (5-HT) signal, which controls oxygen chemoreception and ventilation, was mainly detected in the surface of the body of the larvae, and then shifted to gill filaments of juveniles, showing a transition from cutaneous to branchial respiration. Both larval and juvenile yellow catfish exhibited reduced survival, damaged gill structure, and elevated 5-HT expression after ammonia exposure, as well as upregulated *tph1b*, *slc6a4b*, *scgn* and *lama5* expression with the increased ammonia concentration, indicating the effects on respiratory function via serotonergic regulation. Further transcriptome analysis was conducted in juveniles to identify the differentially expressed genes (DEGs) and thus, to illustrate more detailed responses after ammonia exposure; KEGG enrichment analysis of DEGs indicated the coping strategy shifted from metabolic buffering to metabolic elimination via glutamine synthesis with the increased ammonia level. The qRT-PCR experiment also identified the increased expression of genes involved in the urea cycle—such as *ass1*, *asl* and *glula*—with the increased ammonia level. Considering the potential contributary role of microbiome to gill health, 16S sequencing was conducted on the gill in the control and the 125 mg/L ammonia-exposed group. Ammonia exposure at 125 mg/L induced significant variation in Simpson index and a marked decline in β diversity. Notably, the abundance of opportunistic pathogens such as *Pseudomonadota* increased, while the abundance of *Deinococcota* and *Deinococcus*—which were renowned for exceptional stress resistance capacity—decreased after ammonia exposure. Thus ammonia exposure disrupts the transcriptomic and microecological balance within gill mucosa, which may elevate the risk of pathogenic infection. Overall, our study provided the first evidence of serotonergic regulation on early fish respiration during ammonia exposure, and also offered new theoretical insights into the involvement of microorganisms in ammonia toxicity.

## 1. Introduction

Gill is generally recognized as the main respiratory and gas exchange organ in aquatic animals including teleost fish, which is essentially composed of a highly complex vasculature, surrounded by a high surface area epithelium that only provides a thin barrier between blood and aquatic environment [1]. Espically, the gill epithelium is composed of pavement cells (PVCs), mitochondrionrich cells (MRCs), neuroepithelial cells (NECs) and several other cell types [2]. Unlike the invaginated lungs in terrestrial vertebrates, the evaginated gills in teleost fish are contiguously exposed to the various parameters in water, thus facilitating gas exchange between blood and water [3], but may also frequently challenged by environmental stress and multiple pathogens [4]. Multiple locally resided immune cells have been identified in gill to support the mucosal immune defences, making it a typical mucosal associated lymphoid tissue in fish [5]. However, during early development period when the total body surface area: volume ratio in larval and juvenile fish is high, gas exchange takes place solely by diffusion across nonspecialized body surfaces [6]. Before the onset of active gill respiration at an age of 12–14 d, oxygen supplies in zebrafish (*Danio rerio*) embryos are satisfied via passive diffusion across the skin [7]. Gas exchange of fish evolved from cutaneous respiration to primarily gill respiration. This transition began during the formation of the internal gills and eventually replaced the skin as the primary site for gas exchange. Oxygen uptake of oxygen (O_2_), along with metabolism of organic substrates such as glucose and lipids, is needed to power the biochemical machinery (e.g., in the mitochondria) in cells for body maintenance [8], while carbon dioxide and ammonia are another two gases during gas exchange that are produced in the tissues as waste products of fuel catabolism, and must be removed from the body due to their toxicity [9]. The comprehensive understanding of gas exchange including oxygen, carbon dioxide and ammonia during early development period is important in fishery study.

Recently, the high-density aquaculture system is developed to overcome the limited aquaculture area. However, the accumulated ammonia from feed decomposition and fish excrement within the high-density aquaculture system posed a threat to fish health [10]. In fact, endogenous ammonia synthesis occurs with excess amino acids catabolism, and ~95% of total ammonia in fish blood and internal tissues exists as NH_4_^+^, which is much less toxic than NH_3_ [11]. The endogenous ammonia can be excreted via fish gill branch and renal, which rate in gill of most fish species occupied over 80% of the total nitrogen (ammonia plus urea) [12], as NH_3_ can diffuse through tissues and into a large external aqueous environment. However, the accumulated ammonia in aquaculture system can inhibit the ammonia excretion from fish blood to water environment [13]. Former studies have indicated that the serum ammonia level was significantly elevated after ammonia exposure [14]. High environmental ammonia has been reported to significantly affect the histology structures of gill and other fish tissues. For example, four-finger threadfin (*Eleutheronema tetradactylum*) exhibited substantial gill damage after ammonia nitrogen exposure, including fusion of lamellae and proliferation of chlorine-secreting cells, which acclerated with prolonged exposure period [15]. Female zebrafish exhibited the increased pathological alterations in an ammonia concentration-dependent way, including the hyperplasia of gill lamellae epithelium and the cytoplasmic vacuolization in liver [16]. Besides the pathological changes, high environmental ammonia (HEA) has also been reported to activate multiple hypophysiotropic regulators including the elicitation of increased plasma cortisol [17]. A recent study in rainbow trout (*Oncorhynchus mykiss*) indicated the involvement of 5-hydroxytryptamine (5-HT, also called serotonin) in the hypophysiotropic regulation of hypothalamic-pituitary-interrenal (HPI) axis in response to HEA exposure [18]. 5-HT is a neurotransmitter in the serotonergic neuroendocrine system and plays a key role in many processes including oxygen chemoreception and ventilation regulation, as it is found in the NECs and induced the constriction of gill branchial vasculature [19]. In zebrafish gill, microplastics and copper exposure induced the oxidative stress, apoptosis and serotonergic system changes with the upregulated expression of tryptophan hydroxylase 1a (*tph1a*), the key enzyme involved in 5-HT synthesis and serotonergic activity [20]. However, the effects of ammonia exposure on fish gas exchange during early ontogeny and serotonergic system were rarely evaluated.

Accompanied with the cells and molecules in gill, the resident microbial communities constitute an integral component of innate immune barrier [21]. Early studies have shown that gill microbiota enhance stress resilience through competitive exclusion of pathogens, secretion of antimicrobial metabolites, modulation of mucosal immunity, and enzymatic biotransformation of xenobiotics [22]. Fish gill, as well as gut and skin constituted the so-called mucosa-associated lymphoid tissues (MALT) which serve as the hub connecting microbial signals with host immune responses [23]. Microbiota have been proven to function in the digestion [24], metabolism [25], and immune modulation [26] of teleost. For instance, fish intestine harbors diverse commensal and pathogenic microbial communities [27], which participate in physiological processes ranging from digestive enzyme production to host immune system development [28]. Skin mucosal microbiota were selectively specialized due to the enriched immune factors including antimicrobial peptides and lysozymes within skin mucus and also constitute the first biological barrier protecting fish against environmental stressors. Moreover, recent study in zebrafish and common carp (*Cyprinus carpio*) have also revealed the existance of several ammonia-oxidizing bacteria (AOB) in their gills [29]. In yellow catfish (*Pelteobagrus fulvidraco*), combined ammonia stress and antibiotic exposure significantly altered skin mucus microbial structure and reduced immune indices [30]. Ammonia stress can also alter the mucosal microenvironment and influence microbial colonization selection by activating signaling pathways such as Toll-like receptors (TLR) and nuclear factor-kappa B (NF-κB), thereby inducing the release of pro-inflammatory cytokines [31]. This tripartite “environment-microbe-host” interaction determines the tolerance threshold of fish to ammonia stress.

Yellow catfish is an important freshwater aquaculture fish species in China, while its health and survival were seriously threatened by the accumulated ammonia in the intensive aquaculture system, which may originate from protein-rich feed residues (contributing 60–70% of feed nitrogen to water), fish excretion, and decomposition of organic matter [32]. Former study have reported the ammonia toxicity on multiple yellow catfish tissues [33], and also identified the involvement of microbiota [34] in the resistance against ammonia stress. Recent study with single-cell transcriptomic sequencing has revealed the changes of multiple cell types within fish gill including NECs during stress [35]. Moreover, the intestinal barrier was disrupted by ammonia due to the reduced tight junction protein expression but upregulated inflammatory cytokines [36], accompanied with the reshaped intestinal microbial community [37], while the information about its influence on gill structure and function along with serotonergic regulation is limited. Here, in the present study, the expression pattern of 5-HT in larval yellow catfish were firstly detected via immunofluorence, along with its response during ammonia exposure. Then juvenile yellow catfish were exposed to ammonia, followed by comprehensive analyses of gill tissues encompassing histological examination, 5-HT detection, urea-cycle related gene expression, transcriptomic profiling, and microbiota characterization. All these integrated approaches were used to construct a multi-omics framework delineating the adaptive responses of gill to ammonia challenge. To our acknowledgement, this is the first report about the effects of ammonia exposure on 5-HT expression in both larval and juvenile yellow catfish and on gill microbiota community, which may promote the sustainable development of yellow catfish aquaculture.

## 2. Materials and Methods

### 2.1. Experiment Design

#### 2.1.1. Expt. 1: Early Ontogeny of Yellow Catfish and Ammonia Exposure

Mature broodstock yellow catfish, obtained from broodstock ponds, were selected and transferred to the hatchery. All fish were then acclimated in hatching tanks for 1 day without feeding. Yellow catfish were induced to spawn using a modified hormonal injection protocol based on Chinese patent CN103070120B. Briefly, the selected female fish were injected with luteinizing hormone-releasing hormone analog 2 (LRH-A2) (30 μg/kg fish) and domperidone (DOM) (5 mg/kg fish) and the male fish were injected with same reagents but half dosage. After 12 h, the eggs were stripped into a dry bowl while the testes were removed from the dissected male fish, sheared and grinded thoroughly. Then the eggs were mixed with the grinded testes, and then subjected to debonding treatment with talc powder. After fertilization, the fertilized eggs were transferred to randomized compartments of the incubation tank for further incubation. Larvae came out of the membrane after about 48 h fertilization. During these processes, partial samples of the fertilized eggs and larvae were embedded with optimal cutting temperature compound (OTC). At 2nd day after yellow catfish larvae coming out of the membrane, 900 larvae were distributed into another 9 tanks which were divided into 3 parallel groups to be treated with total ammonia nitrogen (TAN) at 0 mg/L, 10 mg/L, 20 mg/L. The dead larvae were recorded to calculate the survival rate at 24 h, 48 h, and 72 h after ammonia exposure. Partial samples of the ammonia-exposed larvae were also embedded with OTC, used for frozen sectioning.

#### 2.1.2. Expt. 2: Ammonia Exposure in Juvenile Yellow Catfish

120 juvenile yellow catfish with similar body size (weight: 23.18 ± 1.05 g; length: 11.3 ± 0.20 cm) were acclimatized into aquaculture tanks at the Aquaculture Base of Huazhong Agricultural University for one week to ensure they were free from diverse pathogens. Subsequently, the healthy yellow catfish were randomly assigned to three groups (40 fish per group). According the former reported study [38] and our preliminary experiment, juvenile yellow catfish were exposed to different concentrations of ammonium chloride (NH_4_Cl) at 0 mg/L TAN, 25 mg/L TAN, and 125 mg/L TAN, respectively. Here, 25 mg/L TAN is 1/10 of the 96-h ammonia stress LC_50_ (250 mg/L TAN) and represents a stress level encountered during routine high-density culture, while 125 mg/L is half of the 96-h ammonia stress LC_50_ and simulates extreme scenarios. During the exposure period, the water temperature, dissolved oxygen (DO), and pH were maintained at 18.0 ± 0.3 °C, 7.88 ± 0.12 mg/L, and 7.8 ± 0.1, respectively, with continuous aeration. Fish survival and behavioral changes were recorded throughout the experiment, and deceased fish were promptly removed to prevent any impact on the subsequent procedures. After exposure for 96 h, 12 fish from each group were anesthetized with ethyl 3-aminobenzoate methanesulfonate (MS-222) for tissue sampling. During sampling, blood samples were collected from fish caudal vessels with a heparinized syringe. After perfusing with phosphate-buffered saline (PBS), fish gill tissues were separated. Partial segments were embedded with OTC and then frozen for immunofluoerence study, other partial segments were stored in 4% paraformaldehyde (Servicebio, Wuhan, China) for histopathological assay, and all the rest gill samples were frozen in liquid nitrogen and then stored at −80 °C prior to analysis.

### 2.2. Immunofluorescence Assay of Frozen Sections

Immunofluorescence (IF) staining was performed to localize the expression of 5-hydroxytryptamine (5-HT) in fish respiratory tissues. Briefly, the frozen fish eggs, larvae and gill samples were placed into the cryostat microtome (Leica CM1950, Nußloch, Germany) to get the frozen section. After blocking with a suitable buffer (e.g., 5% BSA), the frozen sections were incubated overnight at 4 °C with primary antibodies against 5-HT (SIGMA, 1:500), separately. After washing with PBS for 3 times, the Alexa Fluor 488-conjugated secondary antibody (green fluorescence) were added to the sections and incubated at 4 °C for 2 h. Before mounting, the sections were counterstained with DAPI to stain cell nuclei. Following mounting, sections were visualized under an Olympus BX53 fluorescence microscope. Imaging and subsequent analysis were carried out using iVision-Mac scientific imaging software version 4.0.x.

### 2.3. Preparation of Tissue Sections and H&E Staining

After fixation in 4% paraformaldehyde for 24 h, juvenile yellow catfish gill tissues were cut into small pieces and then dehydrated through a graded ethanol series and cleared with xylene. Then they were embedded in paraffin and sectioned at a thickness of 5 μm. The sections were floated on a water bath, transferred to glass slides, and baked at 65 °C for 2 h. Histological staining was performed using hematoxylin and eosin (H&E). Imaging was conducted using an Olympus DP72 optical microscope (Olympus Corporation, Tokyo, Japan) fitted with a Nikon E600 camera (Melville, NY, USA) and controlled by CellSens Standard software version 4.1.1.

### 2.4. RNA Extraction and qRT-PCR Analysis

Total RNA was extracted from all samples using TRIzol reagent (Invitrogen, Carlsbad, CA, USA) according to the manufacturer’s instructions. RNA purity and concentration were measured with a spectrophotometer of NanoPhotometer NP80 Touch (Implen GmbH, Munich, Germany), and RNA integrity was verified by agarose gel electrophoresis. The A260/A280 of all RNA samples ranges from 1.9 to 2.0, and the strip brightness ratio of distinct 28S/18S bands on 1% agarose gel electrophoresis is 2:1 Equal amounts of qualified RNA (1000 ng) were reverse-transcribed into cDNA using a reverse transcription kit (YEASEN, Shangai, China) as directed by the manufacturer. The synthesized cDNA was diluted to 200 ng/μL and used as the template for quantitative real-time PCR (qRT-PCR). qRT-PCR was performed using EvaGreen 2× qPCR Master Mix (YEASEN, China) under the following conditions: 95 °C for 5 min, followed by 40 cycles of 95 °C for 10 s and 58 °C for 30 s. Primers used in the present study were shown in Appendix A Table A1. The Ct values of target genes were normalized to the geometric mean of two housekeeping genes, ef1α and 18s, and relative expression levels were calculated using the 2^−ΔΔCt^ method. All qRT-PCR data were analyzed with GraphPad Prism 6 software.

### 2.5. RNA-Seq

Gill tissues of yellow catfish subjected to different concentrations of ammonia stress (0 mg/L, 25 mg/L, 125 mg/L) for 96 h were used for transcriptomic analysis. All RNA extraction and quality control sequencing data were performed by Wuhan Zhenyue Biotechnology Co., Ltd., (Wuhan, China), Fastp (https://github.com/OpenGene/fastp, accessed on 1 January 2025) was used to conduct quality control, adapter trimming, quality filtering, and per-read quality pruning of the RNA sequencing data. Subsequently, the clean data of all samples were aligned to the reference genome with accession number: GCF_022655615.1 (https://www.ncbi.nlm.nih.gov/datasets/genome/GCF_022655615.1/, accessed on 1 January 2025). Then, fragments per kilobase million (FPKM) of each gene were calculated based on the length of the gene and the reads count mapped to this gene. Differentially expressed genes (DEGs) were evaluated using DESeq2 with |log2 FoldChange| > 1 and *P*adj < 0.05. ClusterProfiler R software version 4.8.0 packages were used for GO function enrichment and KEGG pathway enrichment analysis of DEGs.

### 2.6. 16S Sequencing

Yellow catfish gill microbiota structure in control (CTRL) group and 125 mg/L ammonia-exposed (AMMO) group were evaluated via 16S sequencing with 6 replicates in each group. Approximately 200 mg of gill tissue was used for the genomic DNA extraction using the TIANGEN Genomic DNA Kit (Tiangen, Beijing, China). Integrity was verified by 1% agarose gel electrophoresis at 5 V/cm for 20 min; concentration and purity were measured with a NanoDrop 2000 spectrophotometer (Thermo Scientific, Wilmington, DE, USA). The following quality criteria were required: dominant band was ≥10 kb, 260/280 ratio 1.8–2.0, 260/230 ratio ≥ 1.5, and Qubit concentration ≥ 30 ng/µL. Then V3–V4 hypervariable region of the 16S rRNA gene was amplified with primers 338F (5′-ACTCCTACGGGAGGCAGCAG-3′) and 806R (5′-GGACTACHVGGGTWTCTAAT-3′). Thermal cycling consisted of 95 °C for 3 min; 29 cycles of 95 °C 30 s, 53 °C 30 s, 72 °C 45 s; and a final extension at 72 °C for 10 min. Amplicons were checked on 2% agarose gels (3 µL load) to confirm correct size, then purified, quantified, and sequenced on the Illumina PE300/PE250 platform (Illumina, San Diego, CA, USA). Raw sequencing reads were quality-controlled using fastp (version 0.23.4; https://github.com/OpenGene/fastp, accessed on 1 January 2025), followed by assembly with FLASH (version 1.2.11; https://ccb.jhu.edu/software/FLASH/, accessed on 1 January 2025). The quality-filtered and assembled sequences were clustered into operational taxonomic units (OTUs) based on 97% sequence identity using USEARCH (version 11; http://drive5.com/usearch/, accessed on 1 January 2025), with chimeric sequences subsequently removed. Taxonomic annotation of OTUs was performed using the RDP Classifier (version 2.11; https://sourceforge.net/projects/rdp-classifier/, accessed on 1 January 2025) against the Silva 138.2/16S_bacteria database, with a confidence threshold of 70%. Alpha- and beta-diversity indices were calculated using Mothur (version 1.30.2) and visualized using R (version 3.3.1). Principal coordinate analysis (PCoA) based on Bray-Curtis distances, combined with ANOSIM/Adonis tests, was employed to assess overall bacterial community differences among samples. All analyses were performed on Majorbio Cloud.

### 2.7. Statistical Analysis of Data

GraphPad Prism 6 was adopted to conduct the statistical analysis of all experimental data. Data of gene expression in ammonia challenge experiment were subjected to one-way analysis of variances (ANOVA), followed by Tukey’ s multiple range tests at the *p* < 0.05 threshold to inspect differences among all the data. Differences in microbiologic population between two groups were analyzed using Student’s *t*-test, and the significance of difference between two groups was labeled by * (*p* < 0.05), ** (*p* < 0.01), and *** (*p* < 0.001). Two-way ANOVA analysis was conducted to evaluate the interactions between ammonia concentration and exposure time on larval survival. Data are representative of at least three independent replicates (mean ± SEM).

## 3. Results

### 3.1. The Distribution of 5-HT in Yellow Catfish During Early Ontogeny

**Figure 1:**
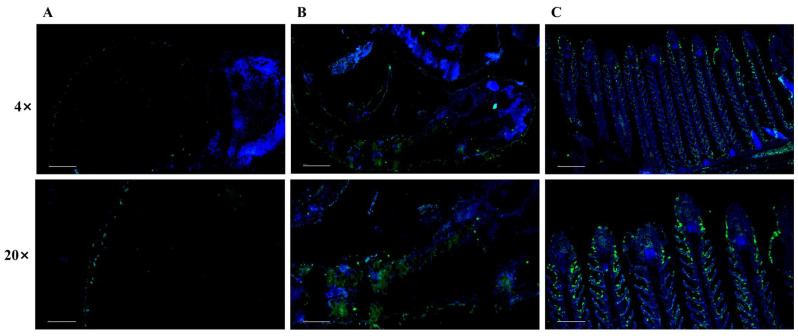
Changes in 5-HT distribution across developmental stages of yellow catfish. Tissues were stained for 5-HT (green) and counterstained for nuclei (blue). Images correspond to (**A**) egg, (**B**) larval, and (**C**) juvenile stages. Scale bar, 4 × 200 μm, 20 × 50 μm.

IF staining with antibody against 5-HT was conducted to reveal its distinct spatial distributions in yellow catfish at various developmental stages. As shown in Figure 1, during the very period, 5-HT positive signals could be detected just along the egg capsules. Furthermore, during the larval stage, the positive signals were distributed throughout the skin of body, indicating the cutaneous respiration in fish larvae. However, in juvenile fish, 5-HT positive signals were predominantly enriched in the epithelial cells of the gill filaments and lamellae. All these results indicated the transition from cutaneous to predominantly branchial respiration.

### 3.2. 5-HT Detection and Survival Rate in Larval Yellow Catfish After Ammonia Exposure

**Figure 2:**
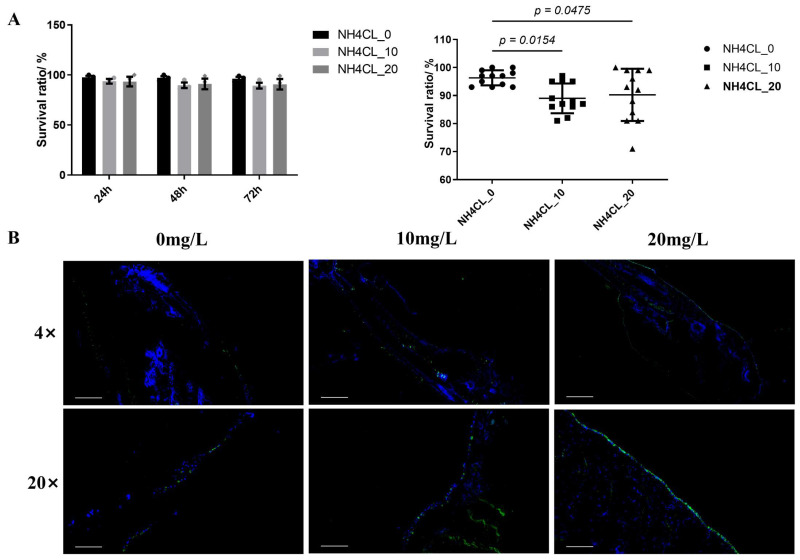
Effects of ammonia nitrogen stress on survival and 5-HT expression in larval yellow catfish. (**A**) Survival rates at different concentrations and time points and survival rates grouped by ammonia concentration (main effect analysis). Values are presented as mean ± SEM (n = 3 replicate tank). (**B**) Representative IF images showing 5-HT expression under corresponding ammonia concentrations, Scale bar, 4 × 200 μm, 20 × 50 μm.

In order to evaluate the effects of ammonia exposure during early developmental period, larval yellow catfish were exposed to ammonia nitrogen stress at different concentrations (0, 10, and 20 mg/L NH_4_Cl). Dead fish were recorded at different durations (24, 48, and 72 h) after ammonia exposure to calculate the survival rates at each time point. Two-way ANOVA analysis revealed no significant interactions between ammonia concentration and exposure time on larval survival (Figure 2A). Subsequently, a significant negative impact on survival rate was observed after ammonia exposure when the effect of ammonia concentration was analyzed separately (*p* < 0.05).

Meanwhile, the distribution of 5-HT containing NEC cells in yellow catfish larvae after ammonia exposure were also detected via IF (Figure 2B). Results in larvae showed that ammonia exposure significantly increased 5-HT distribution, and such increase exhibited an ammonia concentration-dependent response, with higher ammonia concentrations leading to more substantial elevations of 5-HT in larval yellow catfish.

### 3.3. Effect of Ammonia Exposure on Gill Structure and 5-HT Expression of Juvenile Yellow Catfish

**Figure 3:**
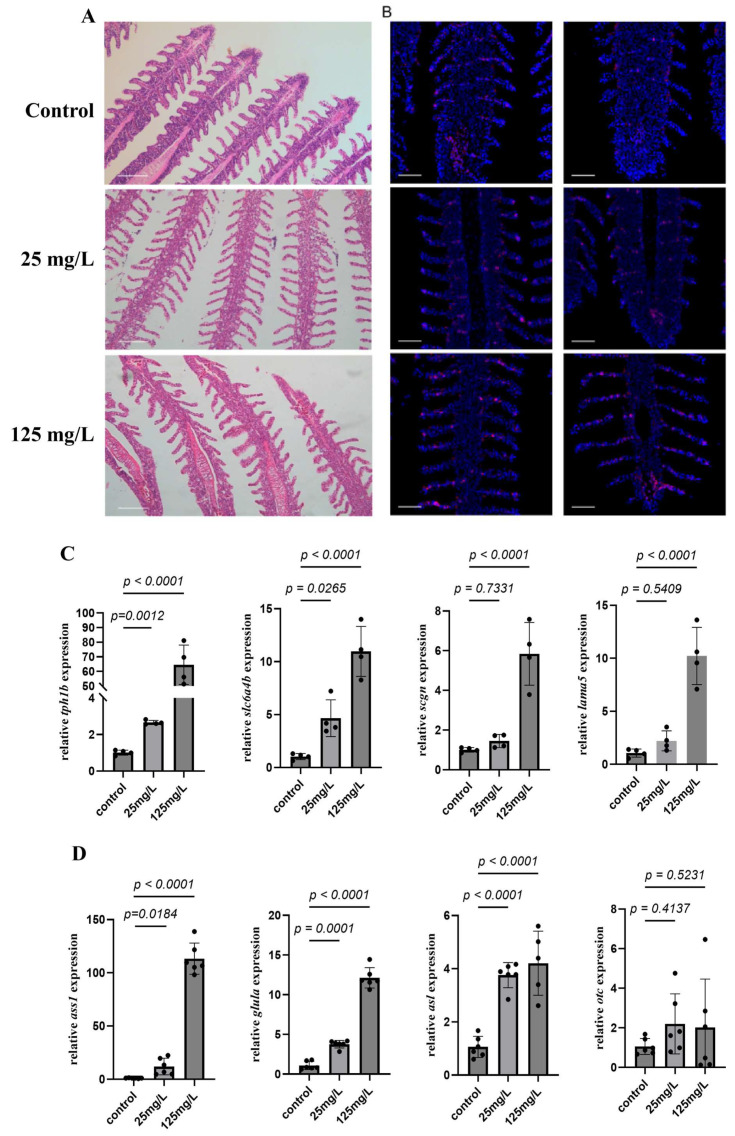
Ammonia exposure induces gill structural damage and alters 5-HT localization. (**A**) H&E staining reveals gill lamellae morphology at various ammonia concentrations. Scale bar, 50 μm. (**B**) Corresponding 5-HT IF signals (purple) with nuclear counterstaining (blue). Scale bar, 50 μm. (**C**) Expression of genes related to 5-HT synthesis (*tph1b*, *slc6a4b*, *scgn* and *lama5*) in yellow catfish gill tissue after exposure to different concentrations of ammonia. (**D**) Expression of genes related to Urea cycle-related genes (*ass1, glula, asl* and *otc*) in yellow catfish gill tissue after exposure to different concentrations of ammonia. Values are presented as mean ± SEM (n = 4 replicate fish).

The gill lamellae of juvenile yellow catfish in control group were tightly arranged, evenly distributed and structurally intact, with the robust filaments. Ammonia exposure significantly changed gill histological structure. As shown in Figure 3A, the gill of yellow catfish exhibited the decreased length of secondary lamina when exposed to ammonia at low concentration. With the increased ammonia exposure dosage, the histological changes further accelerated with the increased vacuolization with the formation of vacuoles within gill tissues and the proliferation of epithelial cells of the gill filaments, along with the occlusion of space between adjacent gill lamellae at high concentration.

Further IF study with antibody against 5-HT was conducted to evaluate the regulatory function of ammonia exposure on fish serotonergic system. Results showed that 5-HT^+^ cells, the chemosensory neuroepithelial cells, is distributed along both primary and secondary lamina of fish gill in control group (Figure 3B). The number and distribution of 5-HT^+^ cells was significantly affected after ammonia exposure. 5-HT^+^ cells’ number significantly increased after ammonia exposure, while 5-HT^+^ cells were detected at the distal gill after 125 mg/L ammonia exposure. To further investigate the regulatory mechanism, RT-qPCR was conducted to evaluate the expression genes involved in the synthesis and transport of 5-HT. As shown in Figure 3C, the expression of tryptophan hydroxylase (*tph1b*), the rate-limiting enzyme for 5-HT synthesis, increased significantly with rising ammonia concentrations (*p* < 0.05). Likewise, the serotonin transporter (*slc6a4b*) was also up-regulated in a dose-dependent manner (*p* < 0.05). Notably, secretagogin (*scgn*) and laminin subunit alpha-5 (*lama5*) remained unchanged under low-level exposure compared with the control (*p* > 0.05), but their transcript levels rose significantly under high-level exposure (*p* < 0.05).

### 3.4. Effects of Different Ammonia Concentrations on Gill Transcriptome in Yellow Catfish

Considering the potential involvement of urea cycle during ammonia stress, the expression levels of urea cycle related genes were detected under ammonia nitrogen stress. As shown in Figure 3D, the expression levels of *ass1*, *glula*, and *asl* significantly increased with the rising ammonia concentration (*p* < 0.05) and exhibited dose-dependent patterns, whereas the expression level of *otc* showed no significant increase (*p* > 0.05).

**Figure 4:**
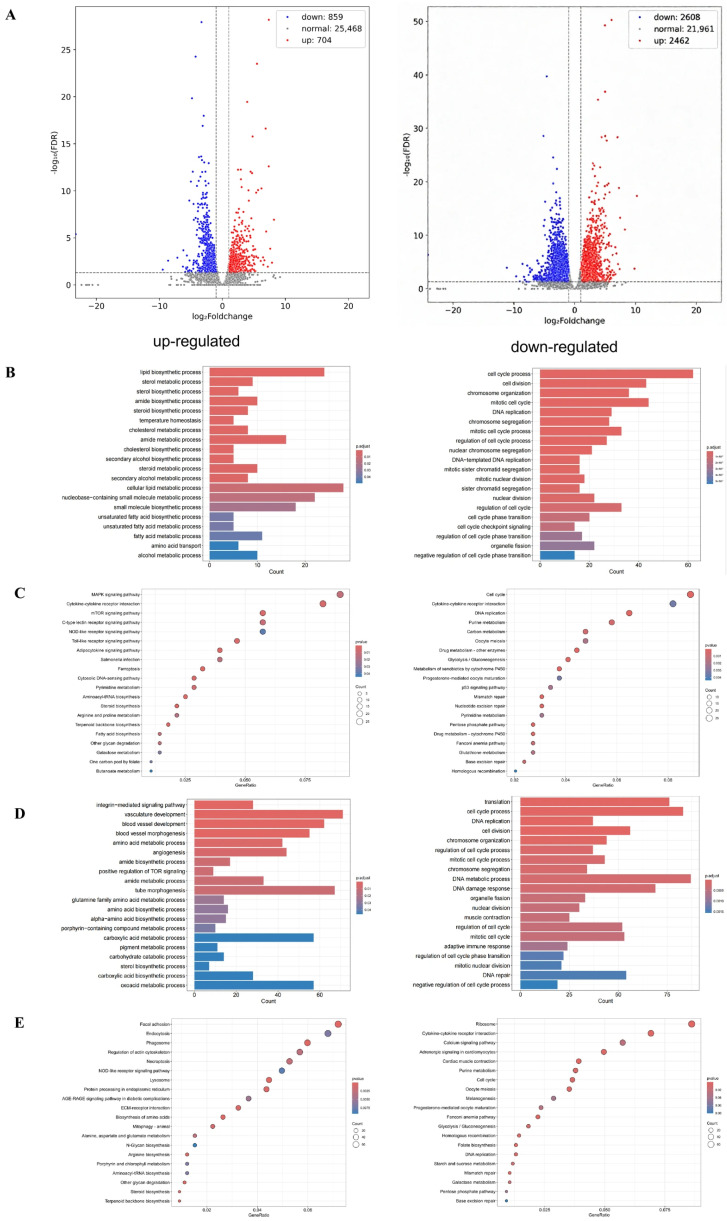
Effects of different ammonia concentrations on gill transcriptome. (**A**) Volcano plot of differentially expressed genes following ammonia exposure at different concentrations. (**B**) GO enrichment bar plot of up- and down-regulated genes under low ammonia exposure. (**C**) KEGG enrichment dot plot of up- and down-regulated genes under low ammonia exposure. (**D**) GO enrichment bar plot of up- and down-regulated genes under high ammonia exposure. (**E**) KEGG enrichment dot plot of up- and down-regulated genes under high ammonia exposure.

In order to elucidate the regulatory mechanism, transcriptomic sequencing was performed on yellow catfish gill in control group, low ammonia (25 mg/L) group and high ammonia (125 mg/L) group. Compared to control group, there were 1563 differentially expressed genes (704 upregulated and 859 downregulated) in low ammonia group. With the increased ammonia exposure dosage, the number of differentially expressed genes increased to 5070 (2462 upregulated and 2608 downregulated) in high ammonia group than control group (Figure 4A). GO enrichment analysis showed the up-regulated lipid biosynthetic process, sterol biosynthetic process, amide biosynthetic process, temperature homeostasis, cellular lipid metabolic process, and small molecule biosynthetic process in low ammonia group, while the down-regulated genes were mainly enriched in cell cycle process, cell division, chromosome organization, mitotic cell cycle, and DNA replication (Figure 4B). KEGG enrichment analysis indicated the upregulated MAPK signaling pathway, Cytokine-cytokine receptor interaction, mTOR signaling pathway, C-type lectin receptor signaling pathway, Toll-like receptor signaling pathway and Ferroptosis, while the downregulated genes were enriched in Cell cycle, DNA replication, Purine metabolism, Carbon metabolism, Oocytemeiosis, Drug metabolism-other enzymes, and so on (Figure 4C). Compared to control group, the upregulated genes in high ammonia group were mainly involved in vasculature development, blood vessel development, blood vessel morphogenesis, glutamine family amino acid metabolic process, angiogenesis, and tube morphogenesis within GO enrichment analysis, while the downregulated genes were enriched in translation, cell cycle process, DNA replication, cell division, DNA metabolic process, DNA damage response, regulation of cell cycle, and mitotic cell cycle (Figure 4D). KEGG enrichment analysis identified the upregulated Focal adhesion, Endocytosis, Phagosome, Regulation of actin cytoskeleton, Necroptosis, NOD-like receptor signaling pathway, and Arginine biosynthesis in high ammonia group, whereas the downregulated genes were enriched in Ribosome, Adrenergic signaling in cardiomyocytes, Purine metabolism, Cell cycle, Oocyte meiosis, and Glycolysis/Gluconeogenesis (Figure 4E).

### 3.5. Effect of Ammonia Exposure on Gill Mcrobial Composition in Yellow Catfish

**Figure 5:**
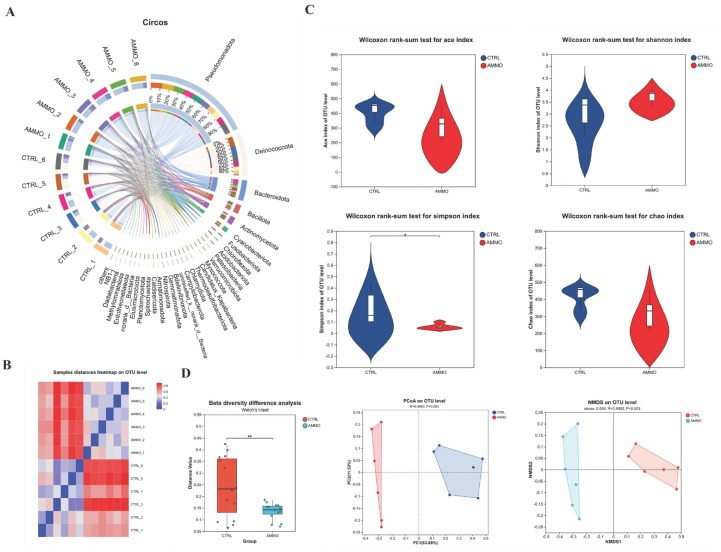
Effects of ammonia exposure on gill microbiota of yellow catfish. (**A**) Cricos plot showing microbial composition across different samples. (**B**) Heatmap illustrating variations among samples. (**C**) α diversity differences (ACE, Chao, Shannon and Simpson index) in gill microbiota. (**D**) β diversity along with PCoA and NMDS analysis in gill microbial community. *, *p *< 0.05; **, *p *< 0.01.

Assembled operational taxonomic units (OTUs) were classified into 45 phyla, 103 classes, 220 orders, 369 families, 772 genera, and 1078 species in yellow catfish gill microbiota of control (CTRL) group and 125 mg/L ammonia-exposed (AMMO) group. The Circos plot illustrated the microbial composition at the phyla level among all test samples (Figure 5A), and the microbial community in CTRL group is dominated by the *Deinococcota*, followed by *Pseudomonadota*, *Bacteroidota, Cyanobacteria* and so on, while *Pseudomonadota* exhibits absolute dominance in the AMMO group, followed by *Bacteroidota* and others. Figure 5B showed the high intra-group reproducibility and significant inter-group variations at the OTU-level. The α diversity within two groups, indicating the species richness and evenness, were determined via the community richness indices (ACE and Chao indices) and community diversity indices (Shannon and Simpson indices). No significant differences were observed in ACE, Chao, or Shannon indices after ammonia exposure, whereas the Simpson index exhibited significant variation between CTRL group and AMMO group (Figure 5C). Principal coordinate analysis (PCoA) based on Bray-Curtis distance revealed clear spatial separation of bacterial communities between the CTRL and AMMO group, with the first two principal coordinates accounting for 75.41% of total variance. Non-metric multidimensional scaling (NMDS) based on weighted UniFrac distance confirmed this finding (stress = 0.034, R = 0.9963, *p* = 0.003). Thus inter-group β diversity analysis revealed a pronounced decline in gill microbiota following 125 mg/L ammonia exposure (Figure 5D).

**Figure 6:**
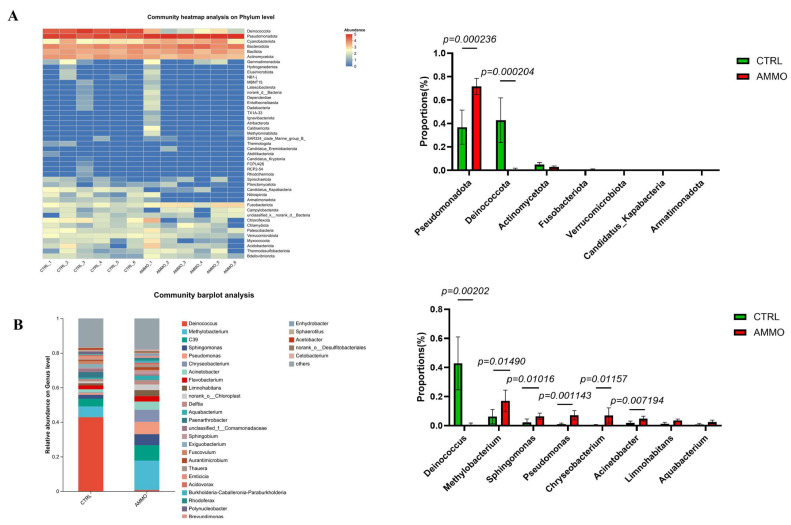
Changes in relative abundance of microbial communities before and after ammonia exposure. Bar plots and Heatmap showing the relative abundance of gill microbial taxa in yellow catfish at different taxonomic levels and the differentially enriched taxa between groups: (**A**) phylum level and (**B**) genus level. Values are presented as mean ± SEM (n = 6 replicate fish).

Moreover, the specific microbial composition at the phylum and genus levels were also systemically evaluated after 125 mg/L ammonia exposure. As shown in Figure 6A, the gill microbiota of yellow catfish was dominated by several predominant phyla at the phylum level. In the control group (CTRL), *Deinococcota* was the dominant phylum with a relative abundance of approximately 43%, followed by *Pseudomonadota* at 37%; while *Bacteroidota* and *Actinomycetota* showed relatively lower abundance. 125 mg/L ammonia exposure markedly altered gill microbiota community, as *Pseudomonadota* became the absolutely dominant phylum, with its relative abundance sharply increasing to 72% (a 1.9-fold increase) while *Deinococcota* abundance dropped substantially to 5% (a 9-fold decrease). Moreover, the relative abundance of *Bacteroidota* abundance increased slightly to 15%. At the genus level, gill microbiota community composition displayed higher diversity, and more pronounced differences in relative abundance of dominant genera were detected between two groups (Figure 6B). In the CTRL group, *Deinococcus* was the absolutely dominant genus with a relative abundance of 43% and *Methylobacterium* ranked second at 7%; while genera including C39 (unclassified), *Sphingomonas*, and *Pseudomonas* showed relatively lower abundance (< 5%). Ammonia exposure drastically restructured microbiota community as the abundance of *Deinococcus* plummeted to 2% while the abundance of *Methylobacterium* significantly increased to 18%, becoming the dominant genus. Moreover, the relative abundance of multiple genera including C39, *Sphingomonas*, *Chryseobacterium*, *Acinetobacter*, and *Flavobacterium* also increased at different degrees.

## 4. Discussion

During fish early development, gas exchange takes place solely by diffusion across nonspecialized body surfaces, while internal gills begin to form and eventually supplant the skin as the major site of gas exchange later in development [39]. The serotonin containing NECs have been reported to serve as peripheral chemical oxygen receptors, capable of detecting hypoxic states in water or blood, and initiating hyperventilation and cardiovascular reflexes to maintain oxygen homeostasis in the internal environment [40]. Further study also identified their distribution along the entire length of the filament but are more concentrated towards the distal half of the filament [41]. Especially, the NECs are found all along the lamellae in goldfish (*C. auratus*) exposed to warm and/or hypoxic water when the interlamellar mass is reduced, while the NECs relocate to the tips of the lamellae as the interlamellar mass fills the space between adjacent filaments when goldfish are exposed to cold and/or normoxic water [42,43]. In both zebrafish and mangrove rivulus (*K. marmoratus*) exposed to sustained hypoxia (28 days and 7 days respectively) there was an increase in serotonergic NEC cell size (hypertrophy) but not density (hyperplasia) [44]. However, zebrafish exposed to hyperoxia for 60 days did exhibit a lower serotonergic NEC density than control fish [45], supporting the role involved in oxygen sensing. Here, in the present study, the expression pattern of 5-HT was evaluated via immunofluerence in yellow catfish pre-larvae, larvae and juveniles. 5-HT positive signals could be detected just along the egg capsules during the very developmental period, while the positive signals were distributed throughout the skin of body during larval stage. In juvenile yellow catfish, 5-HT positive signals were predominantly enriched in the epithelial cells of the gill filaments and lamellae (Figure 1C). All these results indicated the transition from cutaneous to predominantly branchial respiration. Moreover, ammonia exposure significantly increased 5-HT distribution, and such increase exhibited an ammonia concentration-dependent response, with higher ammonia concentrations leading to more substantial elevations of 5-HT in larval yellow catfish. Similarly, the number of 5-HT^+^ cells in juvenile yellow catfish was also significantly increased after ammonia exposure and, especially, ammonia stress at 125 mg/L significantly induced the 5-HT^+^ cells at the distal gill of yellow catfish. All these results indicated the involvement of 5-HT in yellow catfish respiratory tissues during ammonia stress, which is in accordance with results of the involvement of 5-HT in rainbow trout HPI axis in response to HEA exposure [18]. This is also similar to study in mammals that carotid body glomus cells undergo cell proliferation and hypertrophy when mammals are exposed to sustained hypoxia [46]. Further RT-qPCR analysis indicated the increased expression of genes involved in the synthesis and transport of 5-HT in the gill of yellow catfish after ammonia exposure. Early study have shown that norfluoxetine with its parent fluoxetine significantly decreased the RNA expression of serotonin transporters including *slc6a4a* and *slc6a4b* in zebrafish [47], while *scgn* and *lama5*, the representative gene marker of NEC I and NEC II in the gills of juvenile *Eleutheronema tetradactylum* exhibited elevated expression levels under hypoxia [48].

Ammonia has been reported to affect fish at both the individual and tissue level. The ammonia tolerance of fish varied depending on fish species and other water parameters. LC_50_ data gathered from various studies have established safe levels of ammonia forgrowth of marine organism to range between 0.05 and 0.2 mg/L Unlonized Ammonia (UIA)-N, while in sea bass juvenile (*Dicentrarchus labrax*), studies have established that the lethal concentration for 50 percent of the population (96-h LC_50_) was 1.7 mg/L (UIA)-N (40.0 mg/L total ammonia-nitrogen) [49]. In juvenile yellow catfish, former study have showed that the LC_50_ of total ammonia nitrogen (TAN) is 250 mg/L [38], while little information is known about its effects on fish larvae. Our result indicated a significant negative impact on survival rate of larval yellow catfish after ammonia exposure, which is the first study on the effects of ammonia on larval fish. Besides the individual level, ammonia has also been reported to change the histology of multiple fish tissues. For example, in largemouth bass (*Micropterus salmoides*), ammonia exposure induced the swollen hepatocytes, the infiltrated inflammatory cells and hepatic sinus congestion in the liver [50], as well as the thicker epidermal layer, the increased quantity of mucous cells in the skin [51]. Here, our study identified the decreased length of secondary lamina after low ammonia exposure, and the formation of vacuoles and proliferation of epithelial cells of the gill filaments with the increased ammonia dosage. These results are in accordance with previous study with gill hyperplasia and inflammation in yellow catfish [52].

Former study have reported the potential involvement of urea cycle in teleost fish during ammonia challenge [53], thus the mRNA expression of genes involved in urea cycle was evaluated via qRT-PCR. The expression levels of *ass1*, *glula*, and *asl* significantly increased with the rising ammonia concentration (*p* < 0.05) and exhibited dose-dependent patterns, confirming the involvement, which is in accordance with former results [54]. However, the molecular drivers of these structural adaptations and the metabolic strategies employed by gill tissue to cope with ammonia stress needs further evaluation, thus transcriptome sequencing was conducted to illustrate the regulatory mechansim. The DEGs increased from 1563 (704 upregulated and 859 downregulated) to 5070 (2462 upregulated and 2608 downregulated) when ammonia level increased from 25 mg/L to 125 mg/L, indicating the more profound influence by high ammonia level [55]. GO and KEGG enrichment analysis are important in identifying the altered pathway during experimental treatment. MAPK pathway, which is key in transforming extracellular stimuli into intro-cellular responses, is significantly enriched in 25 mg/L ammonia group, which is consistent with results in hybrid grouper (*Epinephelus fuscoguttatus* ♀ × *E. lanceolatus* ♂) [56]. Furthermore, study in rainbow trout liver after ammonia stress showed that carbohydrate metabolism and glycerophospholipid production was inhibited after short-term, while long-term (9 h) ammonia stress inhibited the biosynthesis and degradation of fatty acids, activated pyrimidine metabolism and mismatch repair, lead to DNA strand breakage and cell death, and ultimately caused liver damage [57]. Here in our study, the coping strategy of yellow catfish gill tissue shifted from metabolic buffering to metabolic elimination when ammonia concentration increased from 25 mg/L to 125 mg/L. For example, low ammonia exposure (25 mg/L) resulted in the increased amide synthesis, arginine/proline metabolism, and nitrogenous base metabolism, as well as the sterol and fatty acid biosynthesis. When ammonia concentration increased to 125 mg/L, yellow catfish gill transitioned from a broad-spectrum approach to a focused detoxification reliance on glutamine synthesis, which has been reported to be a key mechanism for ammonia detoxification [58]. Such result is consistent with result in African lungfish (*Protopterus aethiopicus*), with the significantly upregulated *gs* expression during ammonia exposure [59]. Critically, high ammonia exposure triggered several responses including vascular development, angiogenesis, ECM–receptor interactions, and cytoskeletal remodeling, which may increase gill surface area and blood perfusion [60]. Combined with the increased 5-HT expression in gill, such structural adaptation may facilitate physical dilution and excretion of ammonia. Moreover, cells respond accordingly by undergoing various forms of cell death when organisms receive physiological or pathological signals [61]. In the present study, ferroptosis was enriched in yellow catfish gill after 25 mg/L ammonia exposure, while necroptosis were enriched after 125 mg/L ammonia exposure. This is consistent with former study in CEK cells which indicated that ferroptosis was initially activated after HgCl_2_ exposure for 12 h, while necroptosis was activated subsequently at 24 h [62]. Thus ammonia exposure induced ferroptosis and then necroptosis with the increased ammonia level, accompanied with the exacerbated damage on gill structure, the temporal activation of ferroptosis to necroptosis mirrors the metabolic transition from buffering to elimination, suggesting that regulated cell death may clear damaged epithelial cells to facilitate gill remodeling under severe stress.

Previous studies have identified ammonia-induced alterations in tissue structure and metabolic expression in yellow catfish. However, the gill mucosal surface constitutes a complex meta-organism comprising both fish epithelium and resident microbiota [63]. Given that ammonia nitrogen serves as a critical substrate for microbial nitrogen metabolism and transcriptomic data indicated epithelial barrier disruption and ECM remodeling, we hypothesized that ammonia exposure would induce significant dysbiosis of gill microbiota. Therefore, 16S rRNA sequencing was performed to characterize the composition, diversity, and functional potential of gill-associated microbial communities, thereby constructing an integrated host-microbe interaction framework of gill ammonia stress responses. In our study, the Simpson index of gill microbiota exhibited significant variation after 125 mg/L ammonia exposure, showing the declined α diversity with the loss of microbiota richness and evenness. Such result aligns with recent reports in largemouth bass [64] and juvenile cichlids (*Pseudotropheus zebra*) [65], where intestinal α diversity decreased under ammonia stress. Moreover, the β diversity (PCoA and NMDS) dropped markedly after ammonia exposure, demonstrating that ammonia stress not only eroded species richness but also drove the community toward dominance by opportunistic pathogens. Moreover, the relative abundance of specific bacteria both in the phylum and genus level was increased or decreased after ammonia exposure. To be specific, *Pseudomonadota* abundance in the phylum level significantly increased from approximately 37% in the CTRL group to 72% in AMMO group, which was consistent with findings in the intestine of yellow catfish under chronic ammonia stress [41]. Such results demonstrated that ammonia nitrogen stress induced a dramatic shift in gill microbiota from a “*Deinococcota*-dominated” to a “*Pseudomonadota*-dominated” community structure [66]. The bloom of *Pseudomonadota* has been repeatedly observed in nitrogen-enriched aquatic systems, such as in tilapia aquaculture systems where *Pseudomonadota* account for 45–95% of the gill microbiota [67]. Moreover, *Pseudomonas*, *Methylobacterium* and *Acinetobacter* abundance in the genus level were also markedly enriched. *Methylobacterium gregans* DC-1, has been reported to remove 80.9% of total nitrogen within 36h with minimal nitrite accumulation [68], thus the enriched *Methylobacterium* after ammonia exposure may suggest a functional, albeit incomplete, compensation for nitrogen homeostasis. Former study in yellow-catfish skin also detected the elevated *Acinetobacter* abundance after ammonia exposure [69]. Numerous opportunistic pathogens (e.g., *Pseudomonas aeruginosa, P. fluorescens*) is included in *Pseudomonadota* phylum [70] and *Pseudomonas* and *Acinetobacter* are well-recognised opportunist pathogens for fish, thus the dominance of such bacteria may easily invade yellow catfish gill as the host barrier is compromised. On the other hand, the relative abundance of *Deinococcota,* which occupied an absolutely dominant position in the control group (approximately 43%), sharply declined to approximately 5% following ammonia nitrogen stress. Similarly, in the genus level, *Deinococcus* was virtually eradicated in the stressed gills (<1% versus 42% in controls), mirroring the phylum-level trajectory. Previous studies have demonstrated that *Deinococcus* can survive under extreme conditions, attributable to its efficient DNA double-strand break repair mechanisms and unique protein protection strategies [71]. One former study in rice flower carp (*Procypris merus*) identified the significantly positive correlations between *Deinococcus* and antioxidant parameters including CAT activity, MDA levels, and O^2−^ levels [72], thus such decline after ammonia exposure in the present study may decrease the antioxidant capacity and thus resistance capacity of yellow catfish.

## 5. Conclusions

Overall, 5-HT signal can be detected in body surface of yellow catfish larvae and in gill of juveniles which indicated a transition from cutaneous to branchial respiration, while such signal was significantly induced by ammoina exposure. Moreover, ammonia exposure significantly damaged gill histology structure in a dose-dependant manner, along with the transcriptomic response from metabolic buffering to metabolic elimination via glutamine synthesis. Furthermore, the microbiota composition in gill mucus was significantly affected with the increased *Pseudomonadota* but decreased *Deinococcota* after ammonia exposure, thus decreasing fish disease resistance. All these findings provide the first evidence of serotonergic regulation in fish respiration during early ontogeny when exposed to ammonia stress, and also establish a multi-omics framework for understanding host-microbe interactions under environmental stress.

## Data Availability

The original contributions presented in this study are included in the article. Further inquiries can be directed to the corresponding author.

## Appendix A

**Table A1:** All primers used in the experiment.

Primers	Primer Sequence (5′-3′)	Gene ID (NCBI Accession No.)	Product Sizes
*18s*	F: GCAATCCCCAGTCCCAATCA	NC_062539.1	192 bp
	R: GGTCGGCGTCCAACTTCTTA		
*asl*	F: GCGATGCTTCCACACAAACT	NC_062538.1	170 bp
	R: GGATCTCCAGGGAGGATGTCT		
*ass1*	F: GCCAACGCCATCTATGAGGA	NC_062543.1	208 bp
	R: GGATCCTCCAAGGCGAGATG		
*EF1a*	F: GAAGCTTGAGGACAACCCCA	NC_062521.1	250 bp
	R: AGCTGGAATGAGGGTTCACT		
*glula*	F: GCACCTACCAGTCTGAAGGC	NC_062539.1	144 bp
	R: GGTGGTTAGTTTCGGCAGGT		
*lama5*	F: TTCCTGCGCACCAATACACT	NC_062540.1	300 bp
	R: GTGGCAGTTACACTGTTCACAC		
*otc*	F: TACACACAACGTGGAGACCG	NC_062523.1	231 bp
	R: TCCTGGAGGCTTCCCCTTTA		
*scgn*	F: TGGACAACAGCGTGGAGTTT	NC_062520.1	198 bp
	R: CCAAGCGGCCATCTTTGTTC		
*slc6a4b*	F: AATTACTCCCGCTCACCAGC	NC_062526.1	199 bp
	R: GAAGAGTGGCTGTGACCCAA		
*tph1b*	F: CGCCACAGTTCTCATCCACT	NC_062518.1	265 bp
	R: GTGCGTGCTGAAGTTCACTG		
